# Resource allocation in transboundary tuna fisheries: A global analysis

**DOI:** 10.1007/s13280-020-01371-3

**Published:** 2020-09-03

**Authors:** Katherine Seto, Grantly R. Galland, Alice McDonald, Angela Abolhassani, Kamal Azmi, Hussain Sinan, Trent Timmiss, Megan Bailey, Quentin Hanich

**Affiliations:** 1 Australia National Centre for Ocean Resources and Security (ANCORS), University of Wollongong, Building 233, Innovation Campus, Wollongong, NSW 2522 Australia; 2 Nippon Foundation‐Nereus Program, Vancouver, Canada; 3 Environmental Studies Department, University of California at Santa Cruz, Interdisciplinary Sciences Building, Santa Cruz, CA 95064 USA; 4 Galland Consulting, 1714 Summit Pl NW 303, Washington, DC 20009 USA; 5 NRE People, 2 Clara Lane, Casuarina, NSW Australia; 6 Faculty of Law, Institute for Marine and Antarctic Studies (IMAS), Centre for Marine Socioecology (CMS), University of Tasmania, Sandy Bay Campus, Hobart, TAS 7005 Australia; 7 Marine Affairs Program, Dalhousie University, 1355 Oxford St., Halifax, NS B3H 4R2 Canada; 8 Australian Fisheries Management Authority, GPO Box 7051, Canberra, ACT 2601 Australia

**Keywords:** Common pool resource, Equity, Regional fisheries management organization, RFMO

## Abstract

Resource allocation is a fundamental and challenging component of common pool resource governance, particularly transboundary fisheries. We highlight the growing importance of allocation in fisheries governance, comparing approaches of the five tuna Regional Fisheries Management Organizations (tRFMOs). We find all tRFMOs except one have defined resources for allocation and outlined principles to guide allocation based on equity, citizenship, and legitimacy. However, all fall short of applying these principles in assigning fish resources. Most tRFMOs rely on historical catch or effort, while equity principles rarely determine dedicated rights. Further, the current system of annual negotiations reduces certainty, trust, and transparency, counteracting many benefits asserted by rights-based management proponents. We suggest one means of gaining traction may be to shift conversations from allocative rights toward weighting of principles already identified by most tRFMOs. Incorporating principles into resource allocation remains a major opportunity, with important implications for current and future access to fish.

**Electronic supplementary material:**

The online version of this article (10.1007/s13280-020-01371-3) contains supplementary material, which is available to authorized users.

## Introduction

Resource allocation is a fundamental and challenging component of common pool resource (CPR) governance (Ostrom 1990, 2003). In the last three decades, a robust and well-developed literature has considered the conditions under which enduring institutions are able to avert the tragedy of the commons, and sustainably manage CPRs without compromising the resource base (Ostrom 1990; Ostrom et al. 2002; Cox et al. 2010; Agrawal, 2014). However, substantially less research has explored the ways in which those resources are allocated among users, and the implications for fairness, equity, and justice (Agrawal 2003; Albin 2003; Ostrom 2003). In her seminal work, Ostrom emphasizes both these components of good CPR governance, stating that successful CPRs are able to “allocate resource units and at the same time avoid the conflict, uncertainty, and perceived unfairness of a poorly solved assignment problem…” (Ostrom 1990). Transboundary fisheries are a particularly challenging CPR. Several studies have identified characteristics and conditions important to facilitating successful management of CPRs (Wade 1988; Schlager et al. 1994; Agrawal 2001), with some of the most common including small group size, well-defined resource system boundaries, clear user group membership, ease of monitoring and enforcement, and proximity of users to the resource itself (Agrawal 2001). As spatially expansive and remote systems with multinational users exploiting mobile, concealed, multi-species resources, transboundary fisheries meet few if any of these conditions, and are especially difficult to govern.

In an effort to manage these challenging resources, states have established several global frameworks, including the United Nations Convention on the Law of the Sea (UNCLOS), and the United Nations Fish Stocks Agreement (UNFSA), the latter of which formalized the use of Regional Fisheries Management Organizations (RFMOs) for transboundary stock management (United Nations 1982, 1995) (Table 1). To gain access to fishery resources under the mandates of specific RFMO Conventions, states are obliged to cooperate through joining, participating in, or at minimum, applying the conservation and management measures established by the RFMO (see Article 8). Participation includes components like data provision, adhering to compliance criteria, and contributing financially to the RFMO. Within this context, UNFSA identifies allocation as a substantive matter and provides guidance, but grants RFMOs flexibility to determine whether and how to allocate fisheries rights within their jurisdiction (Article 10). Similarly, the FAO Code of Conduct also allows flexibility, but recommends that allocation discussions take into account economic, social, and cultural factors (Paragraph 10, FAO 1995; Hanich and Ota 2013).

**Table 1:** Table to acronyms

CCM	Commission members, cooperating non-members, and participating territories
CCSBT	Convention on the Conservation of Southern Bluefin Tuna
CMM	Conservation and Management Measures
CPR	Common Pool Resource
DWFN	Distant Water Fishing Nation
EEZ	Exclusive Economic Zone
EU	European Union
IATTC	Inter-American Tropical Tuna Commission
ICCAT	International Commission for the Conservation of Atlantic Tunas
IOTC	Indian Ocean Tuna Commission
PNA	Parties to the Nauru Agreement
RFMO	Regional Fisheries Management Organizations
SIDS	Small Island Developing States
TAC	Total Allowable Catch
TAE	Total Allowable Effort
TCAC	Technical Committee on Allocation Criteria
tRFMOs	Tuna-related Regional Fisheries Management Organization
UNCLOS	United Nations Convention on the Law of the Sea
UNFSA	United Nations Fish Stocks Agreement
USD	United States Dollars

Focusing on tuna, early iterations of tuna-related RFMO (tRFMO) conventions emphasized the need for “maintaining the populations of these fishes at a level which will permit maximum sustained catches year after year” (IATTC, 1949) or to “permit the maximum sustainable catch” (ICCAT 1966), but these early conventions make no mention of allocating fish resources to particular actors. However, despite this early emphasis on maintaining abundant fish stocks, tRFMOs are increasingly engaging in allocation processes, considering not only issues of sustainability, but also resource distribution and equity (Bailey et al. 2013a; Abolhassani 2017, 2018; Seto and Hanich 2018). In recent decades, there has been a growing consensus that global resource allocation schemes can, and should, play a role in distributive or corrective justice, aiming to counteract the concentration of resource wealth resultant from colonization and globalization processes (Gupta and Lebel 2010; Pitt et al. 2012). This trend traces some of its roots to the global sustainable development regime, particularly the 1972 United Nations Conference on the Human–Environment in Stockholm, and the subsequent 1987 report of the World Commission on Environment and Development and Rio Declaration, which was adopted by the 1992 United Nations Conference on Environment and Development (UNCED). These major conferences employed language of intra-generational equity, and implicitly or explicitly recognized the unique positions of developing countries with regard to distributing the costs and benefits of resource conservation (Azmi et al. 2016). Other major global CPR regimes to employ language of distributional equity include the shared watercourses (e.g., “equitable and reasonable utilization”) (Bruhacs 1993; United Nations 1997; Kaya 2003; Cinelli 2013; Lankford 2013; Gander 2014), hazardous wastes (e.g., “attend to the specific needs of the developing countries”) (UNEP, 1989; Okereke 2006), and global climate regimes (e.g., “common but differentiated responsibilities”) (Müller et al. 2009; Soltau 2009). With the growth of these discourses, a normative consensus has emerged around the fact that natural resource regimes should, in some way, prioritize the needs and interests of developing or highly resource-dependent states (Van Der Brugt 2012; Hanich 2016).

**Fig. 1:**
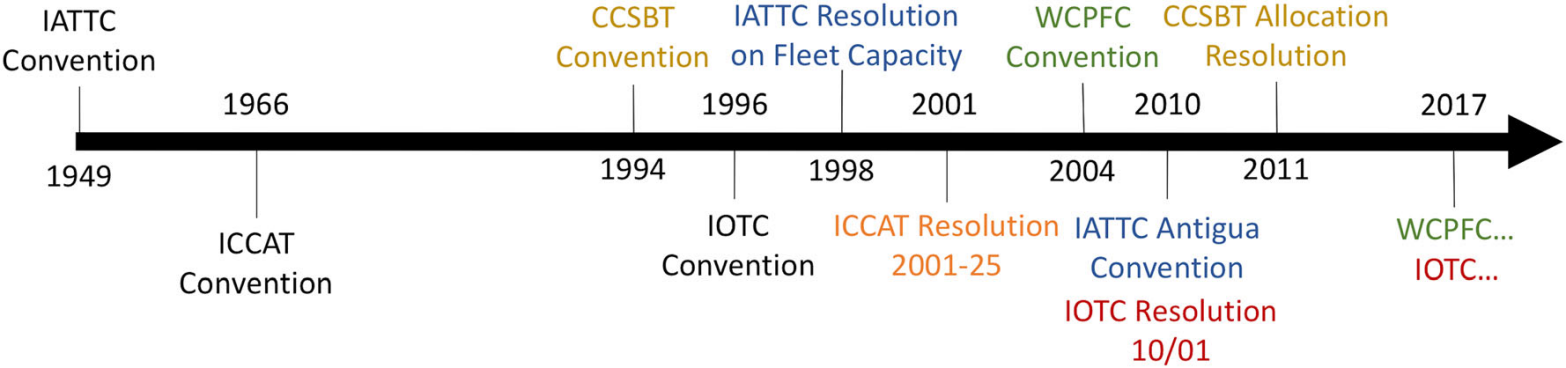
Timeline of initial conventions and emerging allocation language within tRFMOs (by color). WCPFC and IOTC have allocation policymaking processes currently underway

In the context of this increasing concern around not just overall sustainability, but also distribution and equity, these discourses began to impact on fisheries, and specifically transboundary fisheries organizations. Within tRFMOs, this is first observed in the founding text of the Convention on the Conservation of Southern Bluefin Tuna (CCSBT), which explicitly includes in its mandate the ability to allocate fish resources (CCSBT 1994, Article 8(3)a). Following this initial mention, allocation discourse is seen to increase, accelerate, and eventually expand into the language of all five tRFMOs (Fig. 1). This engagement with notions of allocation has implications not only for fish stocks and socio-economic fisheries systems, but also normative implications for the future of how we think about rights and access to resource benefits (Libecap 2010). For example, a key consideration for Libecap is the tradeoff between economic efficiency and equity. Is the goal of allocating a public resource to distribute opportunities to ensure economic efficiency, or is it to distribute opportunities to ensure stewardship or redress historical inequities? These debates are not unique to fisheries (Willis and Bailey 2020), but demonstrate how allocative precedents have lasting implications for the consideration of resource rights.

Allocation has been noted as one of the most fundamental duties of RFMOs, and that without it, achievement of the entire conservation mandate is put at risk (Lodge et al. 2007). More broadly, the certainty and transparency accompanying allocation approaches that are perceived as legitimate are fundamental to the effectiveness of rights-based management (Scott 1996; Árnason 2006). Allocation is not an afterthought to the creation of sustainable limits, it is an integral part of the philosophy behind rights-based management. The primary purposes of this paper are to (1) review the extent to which allocation policies and practices have been implemented in each tRFMO, (2) describe the principles each tRFMO has prioritized to guide that allocation, (3) evaluate the extent to which those principles shape allocative outcomes. In so doing, we consider how the comparison of these policies and practices between tRFMOs may inform ongoing allocation processes. In the context of internationally shared fisheries like tuna, we define allocation as *the assignment of national opportunities to participate in the fishery*. These opportunities may be allocated to individual states or groups of states, and represent a right of extraction, however variously designed. To achieve these purposes, in this paper, we first trace the emergence and growth of allocation discourse in tRFMOs. We assess the degree to which this allocation language has been operationalized through resolutions, measures, and policymaking processes. We consider which of the five tRFMOs have implemented resource allocation approaches, formally or informally, and assess the degree to which these approaches follow the principles outlined by the tRFMO itself. In the absence of explicit, principle-driven allocation, we further assess which principles *implicitly* shape fisheries allocation, noting that the absence of formal approaches by no means precludes de facto allocative outcomes. Finally, we discuss the allocation policies currently under negotiation in two tRFMOs, and the challenges and opportunities each face in implementing comprehensive, principle-driven frameworks for allocating valuable fish resources.

## Materials and methods

The analysis presented here represents a comparative case study of the policies and practices of allocation between all five tuna-related RFMOs. Using content analysis of tRFMO documents and expert consultation, the analysis includes four overall methods. The first two methods below analyze allocation discourses and policymaking, taking historical processes and official document language as the primary subjects of analysis. The second two methods analyze allocation outcomes, taking actual price and quantity of allocated and non-allocated fish as the subject of analysis. Combining these methods enables a comparison of allocative priorities with allocative realities, both within and across tRFMOs.

First, the section entitled “Tracing allocation in tRFMOs: From discourse to reality” uses process tracing to describe the emergence of allocation language and approaches within the historical context of each tRFMO. In establishing an allocation framework for a common pool resource, the general process involves (1) defining and bounding the resource to be allocated and identifying the resource users; (2) identifying the principles upon which the resource system chooses to allocate withdrawal rights (effort or catch quotas); (3) applying those principles to the allocation of the resource; and (4) formalizing that process through a structured, reliable, and assessable mechanism (Ostrom 1990; Pitt et al. 2012). In this section, we provide a brief history of developments within the five tRFMOs, considering where each is situated within this allocation process.

**Fig. 2:**
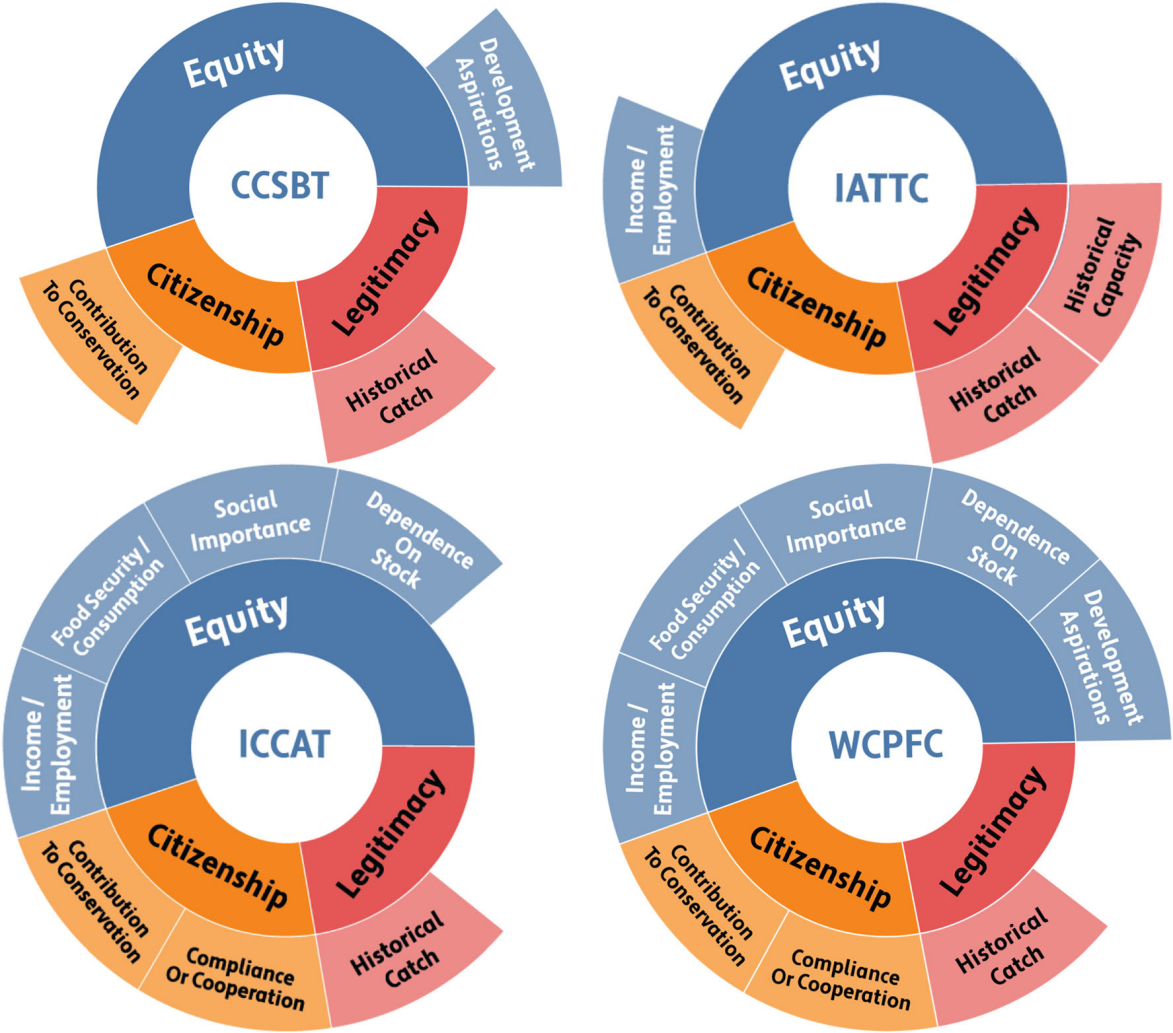
Sunburst plot of allocation principles defined by each tRFMO through resolutions, measures, and policymaking processes. Inner circles represent the categories of principles, and specific principles are listed on the outer circle (WCPFC 2004; CCSBT 1994, 1995, ICCAT 2015b, IATTC 1998)

Second, we conducted a structured summative content analysis (Hsieh and Shannon 2016) of the allocation documents, to compare the allocation principles defined within each tRFMO (Table S1; Fig. 2). Allocation language in each specific tRFMO draws on a number of recurring principles that organizations have agreed should guide rights to fish (e.g., catch or effort history, resource dependence, development status, adjacency). Each of these principles aspires toward certain goals of fairness, equity, or distributional justice, defined along various criteria. Notably, substantial literatures in ethics, philosophy, development economics, environmental justice, political science, and game theory—to name a few—concern themselves with the considerable question of which principles and values should ultimately determine allocation of rights (Shapely 1953; Rawls 1971; Sen, 2000; Okereke 2006; Bailey et al. 2013b). The purpose of this paper is not to evaluate the superiority of these different goals, but to demonstrate that these principles represent the values each tRFMO has declared important in guiding resource rights. Using the qualitative data analysis software MaxQDA (2016), we coded each document for principles that were identified to guide allocation. We further classified each principle based on its intended allocative goal: equity principles emphasize the rights of marginalized actors (e.g., disadvantaged actors or those with greater reliance on the resource); citizenship principles emphasize the rights of those who contribute to cooperative resource management (e.g., through contributions to scientific research or strong records of compliance with RFMO rules); and legitimacy principles stress the rights of those with a historical connection to the fishery (United Nations 1995; Okereke 2006) (Table S1).

**Fig. 3:**
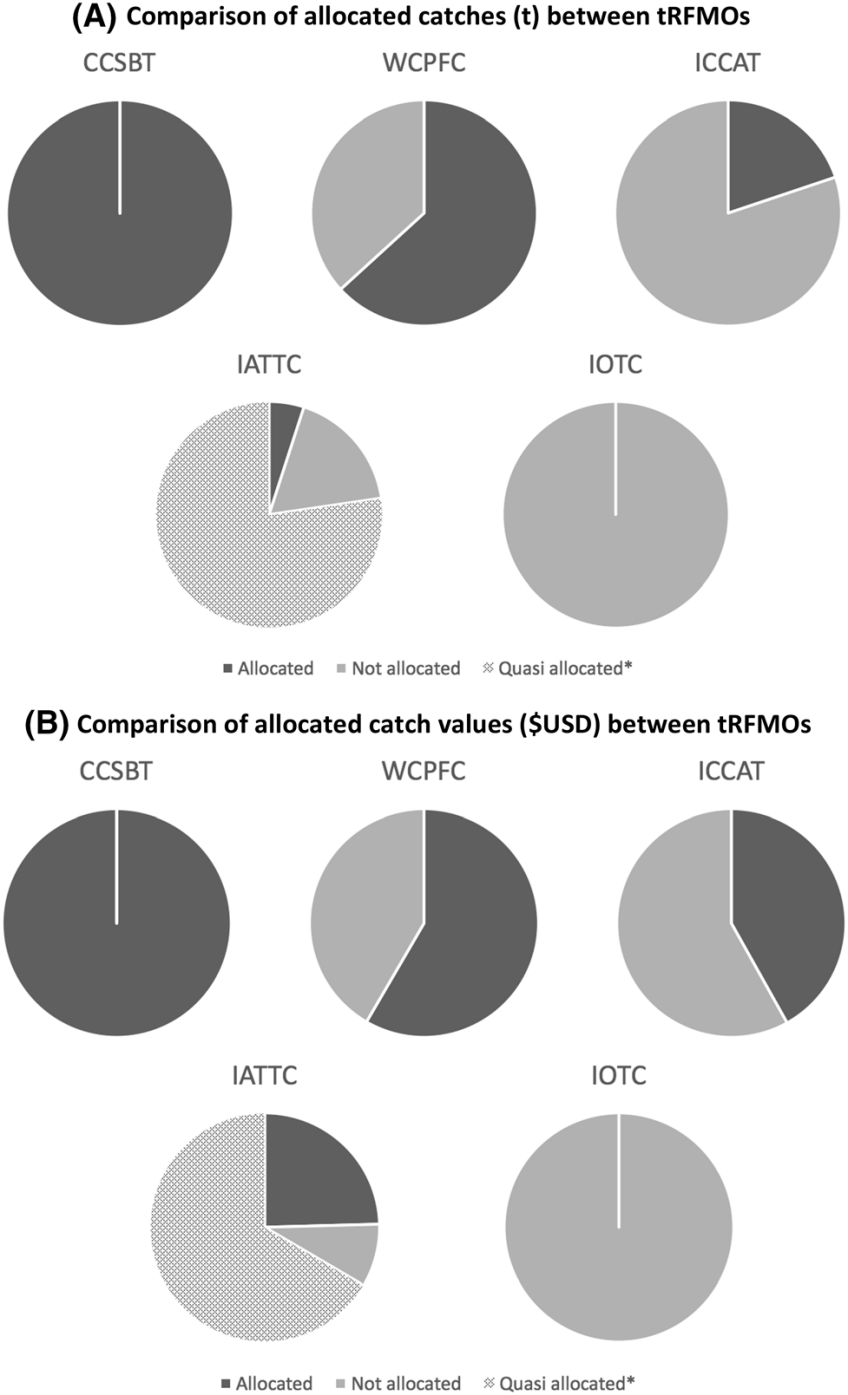
Comparison between tRFMOs of total **a** catches (t) and **b** values (USD) considered allocated vs. non-allocated (IATTC purse seine capacity allocation was considered quasi-allocated). Catch data represent annual catches of all stocks mandated under each tRFMO for the most recent year available (2016 for IATTC, ICCAT, CCSBT 2017 for IOTC, WCPFC), and are not scaled for relative size. Value data are based on 2014 prices and volumes of the seven most commercially important tunas

Third, to examine differences between allocated and non-allocated catch/landings of different tRFMOs, we considered the total annual catches of all stocks (including non-tunas) under the purview of each tRFMO for the most recent year with available data (2016 for IATTC, ICCAT, and CCSBT 2017 for IOTC and WCPFC). We then determined whether each management unit within each tRFMO was considered to be allocated, quasi-allocated, or not allocated based on the definition applied in the introduction and the information and references in Table 2. The pie graphs in Fig. 3a represent allocated proportions within each tRFMO and are not scaled for relative catches between tRFMOs.

**Table 2:** All stocks and management units in all tRFMOs that are considered to be allocated, quasi-allocated, or not allocated for the purposes of this study. Primary references for determining IOTC stocks to be unallocated include yellowfin (IOTC 2019e), skipjack (IOTC 2016), albacore (IOTC 2013; IOTC 2015b); bigeye (IOTC, 2005; Noye and Mfodwo 2012); swordfish (IOTC 2018); and marlins (Indo-Pacific blue, black, striped marlins) (IOTC 2015a)

tRFMO	Stock	Management unit	Considered allocated
ICCAT	E. Atl Bluefin Tuna	Catch by all gears (ICCAT 2017d)	Yes
	W. Atl Bluefin Tuna	Catch by all gears (ICCAT 2017c)	Yes
	S. Atl Swordfish	Catch by all gears (ICCAT 2017b)	Yes
	N. Atl Swordfish	Catch by all gears (ICCAT 2017a)	Yes
	S. Atl Albacore Tuna	Catch by all gears major harvesters (ICCAT 2016d)	Yes
	S. Atl Albacore Tuna	Catch by all gears minor harvesters (ICCAT 2016d)	No
	N. Atl Albacore Tuna	Catch by all gears major harvesters (ICCAT 2016c)	Yes
	N. Atl Albacore Tuna	Catch by all gears minor harvesters (ICCAT 2016c)	No
	Med. Swordfish	Catch by all gears (ICCAT 2016b)	Yes
	Bigeye Tuna	Catch by all gears major harvesters (ICCAT 2016a)	Yes
	Bigeye Tuna	Catch by all gears minor harvesters (ICCAT 2016a)	No
	Blue Marlin	Catch by all gears major harvesters (ICCAT 2015b)	Yes
	Blue Marlin	Catch by all gears minor harvesters (ICCAT 2015b)	No
	White Marlin	Catch by all gears major harvesters (ICCAT 2015b)	Yes
	White Marlin	Catch by all gears minor harvesters (ICCAT 2015b)	No
	All other stocks	Catch by all gears (multiple measures or no management)	No
IATTC	Bigeye Tuna, Yellowfin Tuna, Skipjack Tuna	Purse seine effort (IATTC 2017b)	Quasi
	Bigeye Tuna	Longline catch (IATTC 2017b)	Yes
	Pacific Bluefin Tuna	Catch by all gears (IATTC 2018c)	Yes
	All other stocks	Catch by all gears (multiple measures or no management)	No
CCSBT	S. Bluefin Tuna	Global total allowable catch (TAC) (CCSBT 2017)	Yes
WCPFC	Bigeye Tuna, Yellowfin Tuna, Skipjack Tuna	Purse seine EEZ effort (day) and catch limits (WCPFC 2017a)	Yes
	Bigeye Tuna, Yellowfin Tuna, Skipjack Tuna	Purse seine high seas flag effort (day) limits (WCPFC 2017a)	Yes
	Bigeye Tuna	Bigeye Tuna longline catch flag limits (WCPFC 2017a)	Yes
	Bigeye Tuna, Yellowfin Tuna, Skipjack Tuna	Longline effort (day) limits in EEZs of countries that are party to the PNA Longline VDS (PNA 2016b)	Yes
	Albacore Tuna	Longline Albacore catch taken in EEZs of countries party to the Tokelau Arrangement (FFA 2014)	Yes
	Bigeye Tuna, Yellowfin Tuna, Skipjack Tuna	Purse seine catches in EEZs of countries without a limit listed in CMM 2017-01 Table 2 Att. 1 (WCPFC 2017a)	No
	Bigeye Tuna, Yellowfin Tuna, Skipjack Tuna	Purse seine catches in the high seas by flags without a limit listed in CMM 2017-01 Table 3 Att. 1 (WCPFC 2017a)	No
	Bigeye Tuna, Yellowfin Tuna, Skipjack Tuna	Longline catches in EEZs not captured by a limit in CMM 2017-01 Table 1 Att. 1, nor fall under the PNA longline VDS or Tokelau Arrangement (FFA 2014; PNA 2016b; WCPFC 2017a)	No
	Bigeye Tuna, Yellowfin Tuna, Skipjack Tuna	Longline catches in the high seas by flags without a limit specified in CMM 2017-01 Table 1 Att. 1 (WCPFC 2017a)	No
	Bigeye Tuna, Yellowfin Tuna, Skipjack Tuna	Catches by all gears other than longline and purse seine	No
	All other stocks	Catches by all gears (multiple measures or no management)	No
IOTC	All stocks	Catches by all gears (Negotiations ongoing; Res 10/01 states that allocation criteria should apply to main species)	No

Fourth, to examine differences between allocated and non-allocated values of different tRFMOs, we reexamined the tRFMOs’ allocation practices using available value data from 2014 (Galland et al. 2016; Macfadyen 2016). Price and valuation data were only available for the seven most commercially important tunas (Atlantic bluefin, Pacific bluefin, southern bluefin, bigeye, yellowfin, albacore, and skipjack). Ex-vessel value, as opposed to retail value, was used as this is the more relevant measure for bodies that manage fisheries rather than markets. Once a total value (USD) was calculated for the allocated and not allocated proportions of the catch, we plotted these values (Fig. 3b) to provide a visual comparison to the percent allocated of the total catch under each tRFMO’s mandate (Fig. 3a). In the discussion section, we synthesize the results of process tracing, quantitative, and qualitative comparisons, identifying current gaps and opportunities for allocation within and across tRFMOs.

## Results

**Fig. 4:**
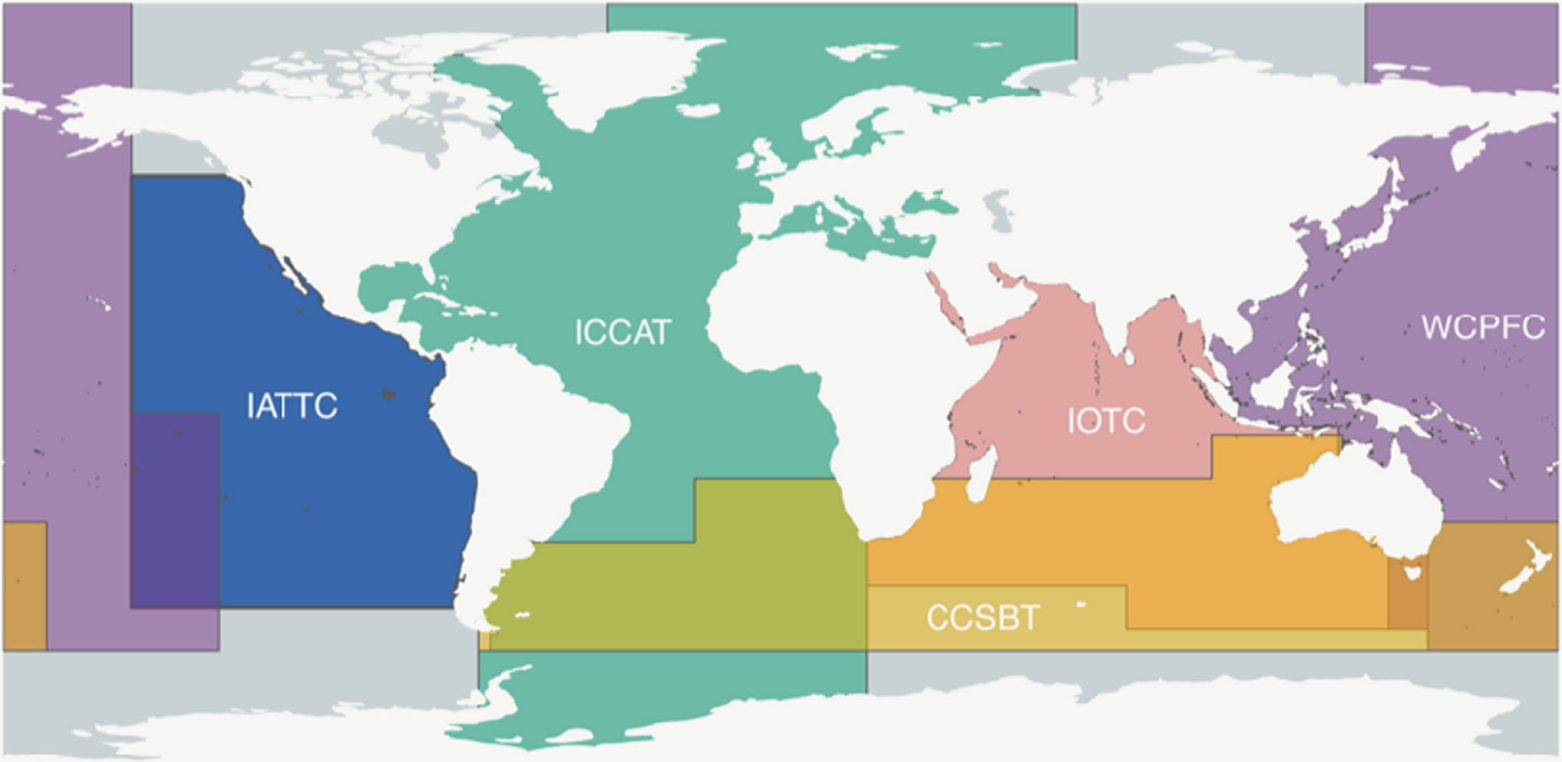
Competence areas of Tuna Regional Fisheries Management Organizations (Image copyright Nate Miller)

In this section, we provide a brief history of developments within the five tRFMOs (Fig. 4), considering where each is situated within this allocation process.

### Inter-American Tropical Tuna Commission (IATTC)

Established in 1949, the Inter-American Tropical Tuna Commission (IATTC) does not include language around limits and allocation within the original convention (IATTC 1949). However, the Antigua Convention, which replaced the 1949 Convention and entered into force in August 2010, states that the organization could, “where necessary, develop criteria for, and make decisions relating to, the allocation of total allowable catch, or total allowable fishing capacity, including carrying capacity, or the level of fishing effort, taking into account all relevant factors” (IATTC 2003, Article 7(1)l). Following this agreement, IATTC developed a management system for the tropical purse seine fleet, where fishing capacity, stock status, and desired management outcomes are combined to determine open and closed seasons for the fishery. “Capacity” is defined by the volume of fish holds (= storage onboard used for catch) and is combined with an estimated or measured catch rate to determine when purse seining should be open or closed. While neither effort nor catch is directly allocated, capacity is frozen at historic levels, leading to a quasi-allocation of available fishing days based on the carrying capacity of each national fleet and the length of the open and closed seasons (Table 2). Early efforts by IATTC to manage in this way began in 1998, with Resolution C-98-11 on Fleet Capacity, which aimed at limiting capacity in response to declining stocks (IATTC 1998). According to this resolution, a capacity limit was allocated to each state, taking into account “various factors including the catch of national fleets during the period 1985–1998; the amount of catch historically taken within the zones where each state exercises sovereignty or national jurisdiction; the landings of tuna in each nation; the contribution of each state to the IATTC conservation program; including the reduction of dolphin mortality; and other factors” (IATTC 1998). Therefore, while Resolution C-98-11 is not specifically an allocation resolution, it effectively outlines the principles that IATTC has chosen to guide the allocation of withdrawal rights (Table 2; Fig. 2).

Since that time, IATTC purse seine fisheries have continued to be managed using a vessel capacity limit—along with associated temporal and area closures—with special considerations granted for historical use, development aspirations, and coastal states (IATTC 1998, 2018a). However, the fishing effort *sinsu stricto* is not allocated, as the open and closed seasons generally apply equivalently to all fleets flying all flags, and total purse seine effort continues to rise (IATTC 2018b). Furthermore, allocation processes are unsystematic, temporary, and directly negotiated between the parties (Mfodwo and Noye 2011). With regard to the longline fisheries targeting bigeye, and for all gears targeting Pacific bluefin, IATTC utilizes a TAC and commensurate allocation scheme. This scheme is also directly negotiated between parties and not determined by systematic weights or formulas (Mfodwo and Noye 2011). In 2017, the European Union (EU) submitted a proposal for the Creation of a Working Group on Allocation of Fishing Opportunities for Tropical Tuna Species (IATTC 2017). According to the Proposal, the Working Group would be tasked with (1) guiding the commission in the development of a TAC, and (2) providing recommendations on criteria for quota allocations of that TAC for tropical tuna in the Eastern Pacific (IATTC 2017). However, the proposal did not receive consensus support—likely due to the asymmetrical benefits to some parties within the current system—and the working group has not yet been formed.

In the overall development and implementation of an allocation framework, IATTC has made ample progress since its inception 70 years ago; however, substantial issues remain. As one of the tRFMOs mandated to manage multi-species fisheries, affected by multiple gears, IATTC has a complex approach to bounding and defining the resource to be allocated (Table 2). While setting the purse seine capacity limit, IATTC defined overall principles that should guide the allocation of effort or catch quota (IATTC 1998), including catch histories, contribution to conservation, and adjacency (Table S1; Fig. 2), but allocation of catch or effort is still subject to annual negotiation.

### International Commission for the Conservation of Atlantic Tunas (ICCAT)

The International Commission for the Conservation of Atlantic Tunas (ICCAT) was established in 1966 and, like IATTC, the original Convention did not include language regarding TACs or allocation of fishing opportunities (ICCAT 1966). The first determination and distribution of a TAC in ICCAT occurred in 1982 for Bluefin tuna stocks, and was directly negotiated based upon “effective monitoring needs, historical catches, and economic factors” (Engler Palma 2010; Mfodwo and Noye 2011). This early allocation mechanism—though controversial—set a precedent within ICCAT toward the establishment and allocation of TACs to manage stocks (Engler Palma 2010). Following this early example, TACs and allocation mechanisms were adopted for North Atlantic Swordfish (1994), Eastern and Mediterranean Bluefin tuna (1998), North Atlantic albacore (2000), South Atlantic swordfish (2002), Bigeye tuna (2011), and South Atlantic albacore (2013) (Engler Palma 2010; ICCAT 2011, 2017). Allocation mechanisms in the early days were most frequently founded on historical catches of flag states, with some exceptions made for development aspirations of coastal states (Engler Palma 2010; Mfodwo and Noye 2011). However, in light of the codification of Exclusive Economic Zones (EEZs) and growing dissatisfaction by coastal developing states with the existing allocations based on historical catches, ICCAT established a working group in 1998 to assess more appropriate allocation criteria (ICCAT 2008; Engler Palma 2010). This process led to the adoption of the non-binding Resolution 2001–2025 (adopted 2001, revised 2015), which outlines allocation criteria and the conditions for applying them (ICCAT 2003, 2015b). ICCAT has four overall criteria for allocation: (1) past or present fishing activities, (2) the status of stocks and fisheries, (3) the status of participants (i.e., various needs and requirements), and (4) compliance with ICCAT Conservation and Management Measures (CMMs) and responsibilities around data submission and research (ICCAT 2003; Henriksen and Hoel 2011). Of particular interest is the third, the Criteria Relating to the Status of the Qualifying Participants, which include explicit considerations for equity and distributional justice, particularly along lines of various forms of historical dependence and socio-economic characteristics (Fig. 2). Although this resolution was met with much enthusiasm from coastal developing states, the application of the criteria is the responsibility of various panels applicable to individual stocks, and implementation has been mixed (Mfodwo and Noye 2011; Serdy 2016a).

Currently, ICCAT has arguably the most extensive system of quotas and allocations of any of the tRFMOs (Fig. 2; Table 2), and resources are defined and bounded based on individual stocks. Furthermore, with the Resolution on Criteria for the Allocation of Fishing Possibilities, ICCAT also has one of the most well-defined sets of principles to allocate fish resources. However, despite the existence of dedicated allocation criteria, allocation mechanisms are not systematic, there are no formulae or weights for application, and many of the principles defined by ICCAT are not detectable in allocative outcomes (Engler Palma 2010; Mfodwo and Noye 2011; ICCAT 2016; Serdy 2016a). Therefore, similar to IATTC, ICCAT has covered substantial ground since its early inception in outlining the resources to be allocated and principles to apply; however, the implementation of those principles remains haphazard. In large part, allocations appear to remain closely linked with historical catches, with considerations for developing and coastal states often taking the form of exemptions from the allocation scheme (ICCAT 2017). Without a systematic approach to applying the criteria, ICCAT TAC allocations are strongly influenced by the positionality of national delegates within annual negotiations, and the principles outlined in Resolution 2001–2025 are not formally or reliably integrated into allocation processes (Serdy 2016a).

### Commission for the Conservation of Southern Bluefin Tuna (CCSBT)

The Commission for the Conservation of Southern Bluefin Tuna (CCSBT) specifically incorporates language around TAC and allocation within Convention text (Article 8(4)). Initial allocation among the original three members predated the Convention, and was based on self-applied catch limits in the 1980s, arising from concerns about the stock, as catches declined by almost two-thirds. In 1995, CCSBT agreed on conditions to be applied when considering allocation to new entrants, based largely on historical catches and conservation concerns (CCSBT 1995; Serdy 2016a) (Fig. 2). However, as the entrants considered were non-parties already fishing the area (Fishing Entity of Taiwan, Indonesia, Republic of Korea, EU), allocating additional quota was intended to represent an increase in official TAC but no actual increase in fish caught (CCSBT 1995). While allocations prior to 2011 were set annually, based on negotiation rather than predefined principles, it is possible to identify some factors that shaped catch limits, including (1) development aspirations and rights of coastal states, (2) technical and economic considerations, (3) contribution to conservation, (4) socio-economic reliance on the fishery, (5) capital investment, 6) historical catch (both by flag and EEZ) (CCSBT 2016).

Since 2011, a management procedure (the Bali Procedure) has been in place, which sets a global TAC for three year periods (CCSBT 2011). The identified TAC is allocated based on the procedures outlined in the Resolution on the Allocation of the Global Total Allowable Catch (adopted 2011, revised 2014 and 2017), which does not clearly outline guiding principles for determining allocations (CCSBT 2017). Current allocations are based strongly on historical catch levels (CCSBT 2016)—subject to political negotiation—and future changes to the TAC will result in relative allocation changes consistent with each member’s or cooperating non-member’s nominal catch percentage (CCSBT 2017). While CCSBT does consider allocation as part of their management procedure, allocation processes are still directly negotiated (Mfodwo and Noye 2011). Negotiated increases to allocations have been primarily based on (1) fleet expansions (e.g., Indonesia) (2) adjustments in catch histories (e.g., South Africa), and (3) consideration of the rights of range (i.e., coastal) states (e.g., South Africa and Indonesia) (CCSBT 2016).

In some ways, CCSBT has the most developed allocation practice of any tRFMO, with quasi-automated allocations occurring triennially based on each state’s nominal catch percentage of the TAC (Table 2). The initial exercise of bounding and defining the resource was likely assisted by CCSBT’s unique situation as the only tRFMO mandated to manage a single stock. Furthermore, its relatively limited membership, catch traceability scheme, and simplicity of fishing methods have undoubtedly played a key role in facilitating the establishment of this allocation framework. However, while CCSBT does define allocation criteria in its convention, these criteria are less developed than other tRFMOs such as ICCAT, and the principles outlined remain highly generalized. Although shaped by the allocation criteria in the convention, the allocation mechanism was not determined by a set of initial principles, but emerged gradually, through complex negotiations and with strong guidance from industry (Pers comm. Glenn Hurry, Head of Australian Delegation to CCSBT 2000–2006; March 12, 2020). To date, CCSBT allocation of southern Bluefin tuna has been almost entirely determined by historical catch levels; however, there are some recent indications that the interests of coastal states and development aspirations are becoming more influential (e.g., increased allocations to South Africa, New Zealand, and Indonesia (Aranda et al. 2010; CCSBT 2014, 2016). While the relative percentage levels outlined in CCSBT create some systematic allocation, these levels are negotiated every 3 years, and are thus more influenced by relative powers of negotiation than the explicit values declared by the tRFMO itself.

### Western and Central Pacific Fisheries Commission (WCPFC)

The Western and Central Pacific Fisheries Commission was established in 2004, and like CCSBT, it includes extensive allocation criteria within its Convention (WCPFC 2004, Article 10(3)a–j) (Figs. 1, 2; Table S1). The issue of allocation was a central component of formative negotiations, revealing a substantial split between the distant water fishing nations (DFWNs), who argued that there should be no differentiation between EEZs and high seas, and Pacific Island states, who strongly asserted that the role of the Commission should be limited to management and allocation of the high seas only (Tarte 2002). As a result, the role of the Commission in allocations is still being negotiated, with the high seas and in-zone (i.e., EEZ) allocation discussions happening separately. No formal allocations have yet been established in WCPFC; however, the Commission has begun a process to allocate limits for high seas fisheries (WCPFC 2017a, 2019), and a complex assemblage of management approaches has emerged, including various forms of limits for fishing in both EEZs and the high seas (Table 2).

The principal CMM that defines these limits is the tropical tuna CMM (adopted 2012, revised regularly up to 2018) (WCPFC 2012, 2018a), which sets out effort and catch restrictions for the two principal fisheries in the WCPFC—the tropical purse seine fishery and the tropical longline fishery. Together, these two fisheries comprise approximately 75% of the tuna catch in the Western and Central Pacific Ocean (WCPO). The limits contained within CMM 2018-01 are not considered formal allocations; in fact, it includes explicit instructions that they “do not confer the allocation of rights to any CCM” (WCPFC 2018a). However, they do represent allocative outcomes, in that they define a country’s (or countries’) allowance to withdraw a specified amount of fisheries resources. As such, for the purpose of this article they will be framed as a form of allocation, although it is recognized that they do not represent an allocation of an ongoing right.

In contrast to other tRFMOs, Pacific Island states have consistently asserted in the WCPFC forum their rights to determine and manage limits on fishing within their EEZs, as prescribed by UNCLOS and UNFSA and protected in the WCPFC Convention. In the utilization of those rights, they have established multi-jurisdictional allocation frameworks for purse seine effort, longline effort, and albacore catch across groups of EEZs (Table 2). These frameworks^1^ all define a total allowable effort or catch limit across the participating jurisdictions and EEZ-specific allocations within that (FFA 2014; PNA 2016a, b). These arrangements apply to all flags fishing in those participating EEZs and cover approximately 54% of all catch in the WCPFC.

As the youngest tRFMO, with some of the most diverse state party membership, WCPFC is uniquely situated with regard to allocative issues. The allocation principles clearly outlined within Article 10(3) of the Convention include principles of sustainability, resource distribution, and equity; however, the current allocative limits under the WCPFC have been primarily driven by historical catch and effort in different zones or by different flags (WCPFC 2005) (Fig. 2). While the allocation frameworks under the Vessel Day Schemes and the Tokelau Arrangement have also included consideration of biomass, equitable sharing, and the development status of fisheries within EEZs, the influence of these criteria on the allocations has been secondary to historical effort and catch.

WCPFC discussions on allocation are expected to advance over the next 3 to 5 years due to two current processes: (1) the commitment made in CMM 2017-01 to allocate high seas purse seine effort and longline catch (WCPFC 2018a, para 28 and 44), and (2) the development of harvest strategies. Although the harvest strategy itself will not address allocations, the impending development of harvest control rules will likely trigger discussions on allocation so that states have greater clarity on how those harvest control rules will be implemented. While there is a formal process for the development of harvest strategies, there is not yet an explicit process for the development of allocations to implement those harvest strategies. The inclusion of timeframes for high seas allocations in CMM 2017-01 represents an important first step in such a process. Within these allocative discussions, Pacific Island states in particular are expected to continue to strongly argue for principles of sustainability and development aspirations to be captured within any allocation outcomes (WCPFC 2004). While WCPFC coastal States have been successful in achieving recognition of their rights to determine limits or allocations applying within their EEZs, moving beyond historical catch and effort in the allocation of high seas fishing opportunities is yet untested.

### Indian Ocean Tuna Commission (IOTC)

The Indian Ocean Tuna Commission (IOTC) was established in 1996, and does not include explicit reference to allocation within the Agreement (IOTC 1993). Discussions on quota allocations began in 2009 in response to a performance review of the IOTC, which recommended that the Commission examine possible advantages and disadvantages to implementing an allocation system (IOTC 2009; Serdy 2016a). That year, the first resolution on allocation was proposed by the EU; however, it relied heavily on the principle of historical catches, and was not adopted by the Commission, as it was deemed unacceptable by multiple parties. Later that year, the IOTC Working Party on Fishing Capacity recommended that input-based allocations (e.g., effort) should be investigated over output-based allocation (e.g., catches); however, this approach was not taken, and IOTC moved in favor of a TAC allocation (Mfodwo and Noye 2011; Noye and Mfodwo 2012).

In March 2010, the IOTC adopted Resolution 10/01, which created an action plan on allocation, involving (1) a technical committee to “discuss allocation criteria for the management of the tuna resources of the Indian Ocean and recommend an allocation quota system or any other relevant measures,” and (2) adoption of “an allocation quota system or any other relevant measure for the yellowfin and bigeye tunas at its plenary session in 2012” (IOTC 2010) (Fig. 1). Since the adoption of Resolution 10/01 in 2010, however, the IOTC has been unable to decide upon allocation criteria, and the subject remains an active area of debate (Abolhassani 2017). The IOTC Technical Committee on Allocation Criteria (TCAC) has convened five meetings since 2011. During this time, IOTC members have submitted several proposals outlining potential quota allocation systems. Since 2016, these proposals have largely reflected the two majority views driving negotiations, represented by the G16, or Group of Like-minded Coastal States in the IOTC, and the EU. Proposals have reflected early agreement within the TCAC on the basic structure of a quota allocation system, including guiding principles, allocation criteria and indicators, a formula to derive allocations, correction factors to adjust allocations, and rules of implementation to govern the use of allocated quota (such as transferring quota) (IOTC 2011).

More recent proposals to the Commission by the G16 and EU have shared common elements but continue to differ on key issues. In 2019, the G16 (IOTC 2019c) and EU (IOTC 2019b) sponsored proposals which both provided for a baseline allocation to all IOTC state parties, consideration of the special requirements of developing states and small island developing states (SIDS), a balance between the rights of Coastal States and DWFNs, and penalties for lack of compliance. The proposals, however, differed substantially in several respects, including the basic structure of their allocation formulas. One ongoing issue IOTC state parties have been unable to resolve is whether historical catch taken within EEZs will be attributed to the coastal state or flag state for the purposes of determining quota allocations. In the G16 proposal, 100% of this catch would be attributed to the relevant coastal state, whereas in the latest edition of the EU proposal, 90% of this catch would be attributed to the relevant flag state, with the remaining 10% being gradually transferred to the coastal state over a decade. DWFNs felt that the G16 proposal represented a drastic change from the current distribution of fishing opportunities, suggesting that the key issue to resolve is the scale and pace of the reattributions and whom these will benefit. A document submitted by the Chair of the TCAC comparing and commenting on the two proposals noted that negotiations on this point presented a ‘very difficult [high] degree of difficulty’ (IOTC 2019g; Sinan and Bailey, in review).

At the most recent meeting of the TCAC in 2019, members were presented with simulations prepared by an external consultant of the allocative outcomes of the two main proposals. While illustrative, simulations of the two allocation proposals did little to significantly advance negotiations. The TCAC noted that the duration of the meeting was not long enough to develop sufficient ‘negotiating momentum’ and ‘resulted in many allocation issues being unresolved’ (IOTC 2019e). In 2019, the Commission agreed to extend the TCAC to a 5 day session in 2020 (IOTC 2019a). Though the TCAC agreed to a 2-year work program in 2018 (IOTC 2018), it remains to be seen how state parties will resolve key issues in future negotiations.

IOTC is the most recent tRFMO to embark upon establishing its allocation framework. Dialogues around allocation have been ongoing at the IOTC for nearly a decade; however, efforts to identify a lasting allocation framework have been recently renewed (Andriamahefazafy et al. 2019). Currently, IOTC bounds and defines the resources on a species by species basis, but limits are only currently set for skipjack and yellowfin tuna (IOTC 2019f). In the case of skipjack tuna, the TAC is not allocated but rather applied as an Olympic race until the limit is reached and adjusted proportionally based on existing catch levels (IOTC 2019f). So far, the limit for skipjack tuna has not been reached and TAC has not been allocated for state parties. In the case for yellowfin tuna, which is overfished and subject to overfishing, state parties have been asked to reduce their catches based on gears they employ compared to 2014 levels (in the case of SIDS, the reduction is based on 2015 levels). Allocation principles are also undefined and currently under negotiation. Like WCPFC, the large number of state parties, food security concerns of coastal states, and historical catch interests of DWFNs all present major challenges to establishing a systematic allocation framework (Andriamahefazafy et al. 2019). Notably, however, in both of the current proposals mentioned above, historical catches remain a dominant factor, allocating between 50% (G16 proposal) and 85% (EU proposal) on historical catches (Andriamahefazafy et al. 2019; IOTC 2019b, c). Moreover, projected shifts in tuna abundance due to climate change, when coupled with coastal states’ development aspirations, further complicate these difficult negotiations. However, recent efforts on the part of coastal states to establish an allocation framework have galvanized the process, and both proposals currently under consideration employ a systematic rule for allocation. One important milestone emerging from the last 2 years of negotiations is the establishment of equitable principles, and their general acceptance among state parties.

## Discussion

In comparing the allocation histories and trajectories of the five tuna-related RFMOs, several trends emerge. First, all tRFMOs with the exception of IOTC have bounded and defined at least some resources for allocation (Fig. 3a). In some cases, these resources represent the entirety of the tRFMO’s mandate (e.g., CCSBT), and in other cases, fish resources intended for allocation are a relatively small proportion of the total stocks managed by the tRFMO (e.g., ICCAT) (Fig. 3a). Furthermore, how tRFMOs define these resources is dramatically different based on the specific stocks, areas, and fishing methods they encompass, contributing to the complexity—and in some cases feasibility—of allocation approaches (Table 2). Notably, when comparing tRFMOs in terms of the *value* of allocated resources (price times volume), for both ICCAT and IATTC, the proportion of the ex-vessel allocated value is higher than the proportion of the total allocated catch (Fig. 3b). In other words, in these tRFMOs, catches of the highest priced stocks tend to be allocated more frequently than catches of lower priced stocks. Two possible explanations for this are that (a) higher prices may lead to increased likelihood of stock depletion, requiring reduced catches and an allocation scheme, or (b) depletion may result in higher prices and reduced catch, increasing the need for an allocation framework. In contrast, WCPFC has a slightly higher percentage of total catch allocated than ex-vessel value, a result likely attributable to the distinct geopolitics of western and central Pacific fisheries. WCPFC has not established allocations for the highest priced species (Pacific bluefin), as it represents only a small fraction of the total WCPO catch (0.3% in 2016) and nearly all the catch is taken by one state in its own waters (Japan) (WCPFC 2016, 2017b). However, a large percentage of the highest volume but lowest priced species (purse seine-caught skipjack) is allocated, with most opportunities allocated to the fishing zone, rather than the flag state (WCPFC 2017a). This management model is not currently replicated by any other RFMO, tuna or otherwise, and has led to a situation where a greater proportion of the ex-vessel value of the resource is shared by the state from which it is extracted (Havice 2013). In both of these cases, by comparing trends between the catches and values of allocated units, it becomes possible to explore the factors (e.g., poor stock status, high price, strong coastal state alliances.) that may increase or decrease the likelihood of allocation.

A second trend is that allocation discourse has played a growing part in the functions of tRFMO management (Fig. 1), and all tRFMOs except IOTC have outlined official principles meant to guide the allocation of these defined resources (Fig. 2; Table 3A). Some of these principles are universal, outlined in the convention, and intended to represent the views of the tRFMO regarding who should receive resource rights (e.g., WCPFC, ICCAT). Others are limited in scope, and specific to individual subsets of resources intended for allocation (e.g., IATTC) (Table 3A). In comparing and contrasting between tRFMOs, these principles emphasize three different sets of values: equity values (emphasizing marginalized groups), citizenship values (emphasizing group cooperation), and legitimacy values (emphasizing historical participation) (Table S1; Fig. 2). Interestingly, all four tRFMOs with allocation principles emphasize all three values, though the specific terms vary. For example, all four explicitly emphasize the principle of historical catch; however, IATTC is the only one to also emphasize historical capacity within its allocation principles. Furthermore, all stress that equity values should shape allocations; however, no single measure of equity is consistently used. The contribution of fish stocks to income and employment was most frequently emphasized; however, other measures of equity (e.g., development status, development aspirations) were also included (Fig. 2). Notably, WCPFC and ICCAT stress multiple equity principles for allocating fish resources; however, CCSBT and IATTC only include one (Fig. 2). Therefore, while allocation discourse is demonstrably growing in tRFMO policy processes—and there is convergence around the values underlying allocation—the diversity of equity principles may impede their implementation relative to the straightforward principle of historical catch.

**Table 3:**
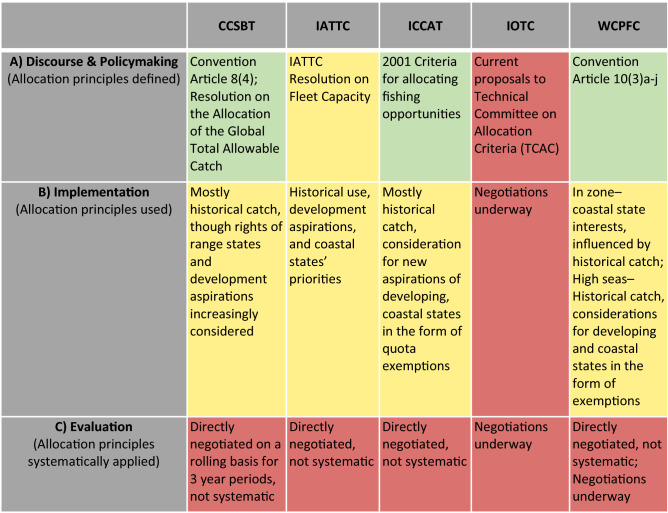
Comparison of the relative status of tRFMOs with regard to A) allocation disclosure & policymaking, B) implementation, and C) evaluation. Green cells indicate that allocation process is complete, yellow is partially complete, and red is incomplete

While these two trends represent substantial accomplishments, all tRFMOs fall short of applying these principles in a structured way toward the equitable allocation of fish resources (Table 3B). To date, most tRFMOs appear to have relied heavily on the principle of historical catch or effort in determining allocations (Lodge and Nandan 1995; Grafton et al. 2006; Engler Palma 2010) (Table 3), and while other principles such as development status or resource dependence have influenced the allocation process, it has primarily been in the form of management exemptions rather than dedicated resource rights (e.g., ICCAT 2015a; WCPFC 2017a). Furthermore, these allocations are determined by direct negotiation between parties, and are thus more shaped by the relative power and positions of individual states and actors rather than the principles selected by each organization (Table 3C). One tRFMO (CCSBT) has a quasi-formalized allocation framework; however, what is formalized within this framework is not the *application* of ranked principles to the allocation of TAC or TAE, but simply the catch percentages allocated among users. While this may increase the certainty that states have regarding their resource rights from year to year, it is still not possible to assess the relative weight or importance given to each allocation principle (Table 3C). There are various strengths and weaknesses inherent in different approaches to weighting (Bailey et al. 2010); however, the act of explicitly weighting values provides the transparency and trust necessary for effective rights-based management. In the absence of explicit, principle-driven allocation, historical catch, and negotiating power become the implicit principles that most strongly shape allocative outcomes (Lodge and Nandan 1995).

By defining and bounding fish resources and identifying resource users, tRFMOs have moved closer to goals of sustainability, and in identifying allocation principles, they have expressed the intention of distributing those resources based on some form of perceived equity (Fig. 2). However, as none have yet created systems for implementing those principles into the act of allocation, this remains a major opportunity for tRFMOs (Table 3c) (Serdy 2016a). The current system of setting yearly (or triennial) allocations based on negotiations between parties reduces certainty for individual states, frequently undermines trust, and ultimately reduces transparency and accountability in allocating rights (Butterworth and Penney 2004; Engler Palma 2010). The current system holds much room for improvement; however, a number of obstacles remain to creating comprehensive allocation systems within the tRFMOs. Differences among states in values, interests, and relative power and influence have implications for which principles they believe should be prioritized in any standardized allocation framework. While this is challenging enough, there is also a real possibility that different principles, expressed in particular ways, may in fact act as mutually exclusive influences on an allocation system (e.g., see Parris and Lee 2009; Soltau 2009). For example, if the principle of historical capacity correlates strongly with high development status, it may be in direct opposition to the principle of development aspirations or dependence on the stock. The role of definitions, weights, and the details of when, where, and to whom an allocation framework applies are also challenging specifics that are both essential preconditions for negotiations and potential snares with the ability to hamstring broadly beneficial policy processes (Bailey et al. 2013a). Decisions on these important details can also be facilitated or constrained by the different policymaking structures and state party compositions specific to each tRFMO (Allen 2010; Nakatsuka 2017). The fact that most tRFMOs rely on (or require) consensus between all parties for a decision regarding allocation is an additional potential hurdle to establishing allocation mechanisms (CCSBT 1994; WCPFC 2004).

Despite these obstacles, both WCPFC and IOTC are currently undertaking major allocation negotiations with implications for fish resource rights for years to come. These negotiations are led by coalitions of SIDS and less developed coastal states, which currently drive efforts toward allocation in both organizations. In IOTC, Maldives and South Africa are leading the allocation negotiation process with a number of coastal states emphasizing equitable allocation that protects the aspirations of developing and least developed countries and recognizes the vulnerability of SIDS. Coastal states are also advocating to attribute historical catches of fish caught in their waters (regardless of fishing flag state), to the coastal states (IOTC 2018). The implications of this could be substantial. Countries that have historically given license to fish in their EEZ would be attributed higher catches; meanwhile, DWFN and coastal states who have fished in those waters are attributed lower catches in the first round of simulations presented to the IOTC member states (IOTC 2019d). In WCPFC, SIDS advocated for a process to allocate high seas fisheries in order to implement sustainable limits through a rights-based management approach that are compatible with the actions being taken in EEZs, thereby transparently ensuring an equitable distribution of benefits (WCPFC 2018b), Article 8 (1)). In both tRFMOs, these coalitions perceive that a standardized allocation system may be the most promising means of ensuring long-term resource rights and implementing those rights based on notions of equity and distributional justice. The outcomes of these allocation processes may remain uncertain for many more years; however, one trend that is clear is that sub-regional bodies and coalitions of like-minded developing states are having a growing influence on the operations of tuna RFMOs (Miller et al. 2014).

## Conclusion

Allocation is a critical component of management. It ensures that access to scarce fish resources is granted based on the priorities and values outlined by the organization, that total catch (or effort) is maintained below levels agreed by the RFMO, and that the organization is perceived as legitimate with strong member buy-in. Allocation of resources can provide developing states the certainty needed to attract investment in fleets and onshore facilities to pursue economic, social, and cultural objectives. Currently, even though most tRFMOs have defined these priorities and values, they have not systematized their application to resource allocation. As such, the actual assignment of resource rights is shaped more by the power, influence, and occasional coercion of individual states, rather than the outlined principles. These assignments are not just important for the year or years to which they apply; these relative allocations can become profoundly valuable, tradeable, and enduring (Serdy 2010, 2016b; Aqorau 2019). They have been proven to shape how resource allocation is perceived and approached for the future, influencing strategies of states that use historical allocations to both constrain and inflate future resource claims (Lodge and Nandan 2005; Libecap 2010). The significant consequences of these allocations underline, all the more, the importance of a principled approach to allocation.

Systematizing the application of allocation principles is not only essential to increase certainty, transparency, and sustainability, but also to shape emerging norms of resource rights and access with purpose and intent. Moving from the current system of opaque allocations based on political negotiations to a systematic and transparent system rooted in the tRFMOs’ principles is not an easy task. We suggest one potential means of gaining traction may be to shift conversations within tRFMOs away from states’ allocative rights and toward weighting of principles that, as we have demonstrated, have already been identified for most tRFMOs (Hanich and Ota 2013). A system of weighted principles would have three clear benefits over the common default approach of using historical catch. First, it would provide a systematic means of incorporating multiple principles, and a method for transparently weighting them according to the priorities of the tRFMO. Second, it would facilitate the quantitative incorporation of those principles (e.g., equity principles) that have historically been underrepresented, through the use of existing indices and metrics (e.g., dependency, development status) (Huang and Słomczyński 2004; Brown et al. 2014; Hirons et al. 2016). Third, a system based on weighted principles and established metrics would not rigidly predetermine annual allocative outcomes (e.g., those based on historical catch) but be responsive to changes in those indices and metrics over time, changing allocative outcomes to continually reflect the initial priorities of the tRFMO. While this would still represent a strongly political negotiation, by reframing around principles, it may be possible to advance toward an allocative *process* without becoming mired in the debates around allocative *outcomes*. Although no tRFMOs have currently instituted this kind of systematic allocative framework, there are important precedents for establishing these processes at sub-regional levels (Aqorau et al. 2018). The process of creating international rules for resource allocation has always been, and will necessarily be, slow. However, there is currently substantial momentum—and unique opportunities—to establish these frameworks for several global tuna stocks, shifting the ways we think about rights and access to fish both now and in the future.

## Electronic supplementary material

Below is the link to the electronic supplementary material.

Supplementary material 1 (PDF 70 kb)

## Footnotes

1 The Palau Arrangement for the Management of the Western Pacific Fishery Management Scheme (Purse Seine Vessel Day Scheme); the Palau Arrangement for the Management of the Western Pacific Tuna Fishery—Management Scheme (the PNA Longline Vessel Day Scheme); and the Tokelau Arrangement for the Management of the South Pacific Albacore Fishery.
