# Supplementary material for: Resource allocation in transboundary tuna fisheries: A global analysis
Source: Ambio. 2020 Sep 3;50(1):242–59. doi: 10.1007/s13280-020-01371-3 (PMC7708539; doi:10.1007/s13280-020-01371-3)
Supplement: Supplementary file 1 — Supplementary material 1 (PDF 70 kb) [file 13280_2020_1371_MOESM1_ESM.pdf]

Ambio

Electronic Supplementary Material
This supplementary material has not been peer reviewed.

Title: **Resource allocation in transboundary tuna fisheries: a global analysis**

Authors: Katherine Seto, Grantly R. Galland, Alice McDonald, Angela Abolhassani, Kamal Azmi, Hussain Sinan, Trent Timmiss, Megan Bailey, Quentin Hanich

Allocative goal	Allocation principle	RFMO	Reference	Text excerpt
Citizenship	Contribution to conservation	WCPFC	WCPFC. 2004. Convention on the Conservation and Management of Highly Migratory Fish Stocks in the Western and Central Pacific Ocean.	the respective contributions of participants to conservation and management of the stocks, including the provision by them of accurate data and their contribution to the conduct of scientific research in the Convention Area;
Citizenship	Compliance or cooperation	WCPFC	WCPFC. 2004. Convention on the Conservation and Management of Highly Migratory Fish Stocks in the Western and Central Pacific Ocean.	the record of compliance by the participants with conservation and management measures;
Citizenship	Contribution to conservation	CCSBT	CCSBT. 1994. Convention for the Conservation of Southern Bluefin Tuna.	contribution of each Party to conservation and enhancement of, and scientific research on, southern bluefin tuna;
Citizenship	Contribution to conservation	ICCAT	ICCAT. 2015b. 15-13 Resolution by ICCAT on Criteria for the Allocation of Fishing Possibilities.	Have the ability to apply the conservation and management measures of ICCAT, to collect and to provide accurate data for the relevant resources and, taking into account their respective capacities, to conduct scientific research on those resources.
Citizenship	Compliance or cooperation	ICCAT	ICCAT. 2015b. 15-13 Resolution by ICCAT on Criteria for the Allocation of Fishing Possibilities.	The record of compliance or cooperation by qualifying participants with ICCAT's conservation and management measures
Citizenship	Compliance or cooperation	ICCAT	ICCAT. 2015b. 15-13 Resolution by ICCAT on Criteria for the Allocation of Fishing Possibilities.	The exercise of responsibilities concerning the vessels under the jurisdiction of qualifying participants.
Citizenship	Contribution to conservation	ICCAT	ICCAT. 2015b. 15-13 Resolution by ICCAT on Criteria for the Allocation of Fishing Possibilities.	The contribution of qualifying participants to conservation and management of the stocks, to the collection and provision of accurate data required by ICCAT and, taking into account their respective capacities, to the conduct of scientific research on the stocks.
Citizenship	Contribution to conservation	ICCAT	ICCAT. 2015b. 15-13 Resolution by ICCAT on Criteria for the Allocation of Fishing Possibilities.	application of the allocation criteria should take into account the contributions to conservation made by qualifying participants necessary to conserve, manage, restore or rebuild fish stocks in accordance with the objective of the Convention.
Citizenship	Contribution to conservation	IATTC	IATTC. 1998. C-98-11 Resolution on Fleet Capacity: 1–2.	contribution of each state to the IATTC conservation program
Equity	Contribution to food security/consumption	WCPFC	WCPFC. 2004. Convention on the Conservation and Management of Highly Migratory Fish Stocks in the Western and Central Pacific Ocean.	extent of the catch being utilized for domestic consumption;
Equity	Dependence on stock	WCPFC	WCPFC. 2004. Convention on the Conservation and Management of Highly Migratory Fish Stocks in the Western and Central Pacific Ocean.	needs of small island developing States, and territories and possessions, in the Convention Area whose economies, food supplies and livelihoods are overwhelmingly dependent on the exploitation of marine living resources;
Equity	Contribution to social importance	WCPFC	WCPFC. 2004. Convention on the Conservation and Management of Highly Migratory Fish Stocks in the Western and Central Pacific Ocean.	needs of small island developing States, and territories and possessions, in the Convention Area whose economies, food supplies and livelihoods are overwhelmingly dependent on the exploitation of marine living resources;
Equity	Contribution to food security/consumption	WCPFC	WCPFC. 2004. Convention on the Conservation and Management of Highly Migratory Fish Stocks in the Western and Central Pacific Ocean.	needs of small island developing States, and territories and possessions, in the Convention Area whose economies, food supplies and livelihoods are overwhelmingly dependent on the exploitation of marine living resources;
Equity	Contribution to income and employment	WCPFC	WCPFC. 2004. Convention on the Conservation and Management of Highly Migratory Fish Stocks in the Western and Central Pacific Ocean.	needs of small island developing States, and territories and possessions, in the Convention Area whose economies, food supplies and livelihoods are overwhelmingly dependent on the exploitation of marine living resources;
Equity	Development aspirations	WCPFC	WCPFC. 2004. Convention on the Conservation and Management of Highly Migratory Fish Stocks in the Western and Central Pacific Ocean.	the fishing interests and aspirations of coastal States, particularly small island developing States, and territories and possessions, in whose areas of national jurisdiction the stocks also occur.
Equity	Development aspirations	CCSBT	CCSBT. 1994. Convention for the Conservation of Southern Bluefin Tuna.	those which have southern bluefin tuna fisheries under development;
Equity	Dependence on stock	ICCAT	ICCAT. 2015b. 15-13 Resolution by ICCAT on Criteria for the Allocation of Fishing Possibilities.	The needs of the coastal fishing communities which are dependent mainly on fishing for the stocks.
Equity	Dependence on stock	ICCAT	ICCAT. 2015b. 15-13 Resolution by ICCAT on Criteria for the Allocation of Fishing Possibilities.	socio-economic contribution of the fisheries for stocks regulated by ICCAT to the developing States, especially small island developing States and developing territories1 from the region
Equity	Dependence on stock	ICCAT	ICCAT. 2015b. 15-13 Resolution by ICCAT on Criteria for the Allocation of Fishing Possibilities.	respective dependence on the stock(s) of the coastal States, and of the other States that fish species regulated by ICCAT
Equity	Contribution to social importance	ICCAT	ICCAT. 2015b. 15-13 Resolution by ICCAT on Criteria for the Allocation of Fishing Possibilities.	economic and/or social importance of the fishery for qualifying participants whose fishing vessels have habitually participated in the fishery in the Convention area
Equity	Contribution to income and employment	ICCAT	ICCAT. 2015b. 15-13 Resolution by ICCAT on Criteria for the Allocation of Fishing Possibilities.	economic and/or social importance of the fishery for qualifying participants whose fishing vessels have habitually participated in the fishery in the Convention area
Equity	Contribution to food security/consumption	ICCAT	ICCAT. 2015b. 15-13 Resolution by ICCAT on Criteria for the Allocation of Fishing Possibilities.	contribution of the fisheries for the stocks regulated by ICCAT to the national food security/needs, domestic consumption,
Equity	Contribution to income and employment	ICCAT	ICCAT. 2015b. 15-13 Resolution by ICCAT on Criteria for the Allocation of Fishing Possibilities.	contribution of the fisheries for the stocks regulated by ICCAT to the national food security/needs, domestic consumption, income resulting from exports, and employment of qualifying participants.
Equity	Contribution to income and employment	IATTC	IATTC. 1998. C-98-11 Resolution on Fleet Capacity: 1–2.	landings of tuna in each nation
Legitimacy	Historical catch	WCPFC	WCPFC. 2004. Convention on the Conservation and Management of Highly Migratory Fish Stocks in the Western and Central Pacific Ocean.	the historic catch in an area;
Legitimacy	Historical catch	CCSBT	CCSBT. 1995. Report of the Second Annual Meeting. Tokyo: 1–21.	The quota allocation to new entrants including cooperative Parties will be calculated based on the past catch records of the new entrant prior to the signature of the Convention for the Conservation of Southern Bluefin Tuna by the present three Parties in 1993
Legitimacy	Historical catch	ICCAT	ICCAT. 2015b. 15-13 Resolution by ICCAT on Criteria for the Allocation of Fishing Possibilities.	Historical catches of qualifying participants.
Legitimacy	Historical capacity	IATTC	IATTC. 1998. C-98-11 Resolution on Fleet Capacity: 1–2.	with significant installed processing capacity
Legitimacy	Historical catch	IATTC	IATTC. 1998. C-98-11 Resolution on Fleet Capacity: 1–2.	catch of national fleets during the period 1985-1998

Table S1. Summary of allocation documents and text excerpts comprising the structured summative content analysis of allocation principles and their overall allocative goals.
